# S02-5 Physical inactivity in nine Eastern European and Central Asian countries: results from the WHO STEPwise approach to NCD risk factor surveillance

**DOI:** 10.1093/eurpub/ckac093.010

**Published:** 2022-08-29

**Authors:** Stephen Whiting, Romeu Mendes, Karim Abu-Omar, Peter Gelius, Anna Crispo, Karen McColl, Phillipa Simmonds, Natalia Fedkina, Dianne Andreasyan, Hagverdiyev Gahraman, Tatyana Migal, Lela Sturua, Galina Obreja, Zulfinissio Abdurakhmanova, Ibraeva Nurgul Saparkulovna, Toker Erguder, Banu Ekinci, Bekir Keskinkilic, Shukhrat Shukurov, Rustam Yuldashev, Nino Berdzuli, Ivo Rakovac, Joao Breda

**Affiliations:** 1 WHO European Office for the Prevention and Control of Noncommunicable Diseases, Moscow, Russian Federation; 2 EPIUnit—Instituto de Saúde Pública, Universidade do Porto, Porto, Portugal; 3 Department of Sport Science and Sport, FAU, Erlangen, Germany; 4 Epidemiology and Biostatistics Unit, Istituto Nazionale Tumori-IRCCS-Fondazione G. Pascale, Napoli, Italy; 5 National Institute of Health, National Health Information Analytic Centre, National Institute of Health, Yerevan, Armenia; 6 Public Health and Reforms Center of the Ministry of Health, Baku, Azerbaijan; 7 Department of Health Care Organization of the Ministry of Health, Minsk, Belarus; 8 National Center for Disease Control and Public Health, Tbilisi, Georgia; 9 Department of Social Medicine and Health Management, State University of Medicine and Pharmacy, Chisinau, Republic of Moldova; 10 Republican Nutrition Center, Dushanbe, Tajikistan; 11 Organization of Medical Care and Medicines Policy, Bishkek, Kyrgyzstan; 12 WHO Country Office in Turkey, Ankara, Turkey; 13 General Directorate of Public Health, Ministry of Health, Ankara, Turkey; 14 Central Bureau for the implementation of the Health-3 project, Tashkent, Uzbekistan; 15 Division of Country Health Programmes, WHO Regional Office for Europe, Copenhagen, Denmark

**Keywords:** Physical activity, surveillance, NCDs, Policy, Health

## Abstract

**Background:**

Physical inactivity is a major risk factor for noncommunicable diseases. This paper explores patterns of physical inactivity in Armenia, Azerbaijan, Belarus, Georgia, Kyrgyzstan, Moldova, Tajikistan, Türkiye and Uzbekistan.

**Methods:**

Nationally-representative data were collected through the WHO STEPwise survey of noncommunicable disease risk factors, which utilizes the Global Physical Activity Questionnaire (GPAQ) to estimate population physical inactivity.

**Results:**

The prevalence of physical inactivity varied across the region, from 11.4 to 44.7%, and was higher among women than men in all countries except for Armenia, Belarus and Moldova. In most countries, the highest proportion of physical activity levels were registered during work time and appeared to vary according to the countries’ level of development. For both sexes and across all populations, time spent on leisure or recreational physical activity was low.

**Conclusions:**

These results have important implications for policy, including actions to promote active travel and leisure-time physical activity as countries develop economically. Investments in workplace physical activity programmes and infrastructure are needed as populations transition to sedentary from more physically active occupations. The results reiterate the need for multisectoral policies, programmes and interventions to promote physical activity tailored to the local context.

